# COVID-19 Vaccine Acceptors, Refusers, and the Moveable Middle: A Qualitative Study from Central Texas

**DOI:** 10.3390/vaccines10101739

**Published:** 2022-10-18

**Authors:** John R. Litaker, Carlos Lopez Bray, Naomi Tamez, Wesley Durkalski, Richard Taylor

**Affiliations:** 1 Office of Population Health and Science, The Litaker Group, LLC, Austin, TX 78716, USA; 2 Office of Population Health, Sendero Health Plans, Inc., Austin, TX 78741, USA; 3 Office of the Chief Executive Officer, Sendero Health Plans, Inc., Austin, TX 78741, USA; 4 Undergraduate Public Health Program, University of Texas at Austin, Austin, TX 78712, USA

**Keywords:** COVID-19, vaccine hesitancy, vaccine acceptors, vaccine refusers, moveable middle, sendero health plans

## Abstract

COVID-19 has caused excessive morbidity and mortality worldwide. COVID-19 vaccines, including the two mRNA vaccines, were developed to help mitigate COVID-19 and to move society towards herd immunity. Despite the strong efficacy and effectiveness profile of these vaccines, there remains a degree of vaccine hesitancy among the population. To better understand hesitancy associated with COVID-19 vaccines and to delineate between those who are vaccine acceptors, vaccine refusers, and the moveable middle, we conducted a cross-sectional survey to understand respondents’ decision to receive, or not, a COVID-19 vaccine at the onset of mRNA vaccine availability in Central Texas. A total of 737 individuals responded, with 685 responses classified to one of eight domains: A: End to the Pandemic (*n* = 48); B: Trust in Medical Community (*n* = 27); C: Illness-Focused Perceptions (*n* = 331); D: Social Motivation (*n* = 54); E: Vaccine-Focused Perceptions (*n* = 183); F: Knowledge Gap (*n* = 14); G: Underlying Health Concern (*n* = 9); and H: Undecided (*n* = 19). Vaccine acceptors (*n* = 535) were primarily represented in domains A–E, while vaccine refusers (*n* = 26) were primarily represented in domains C, E, G, and H. The moveable middle (*n* = 124) was primarily represented by domains C–H. These findings show clear delineations between vaccine acceptors, vaccine refusers, and the moveable middle across eight domains that can assist public health professionals in addressing vaccine hesitancy.

## 1. Introduction

Vaccine-induced herd immunity continues to represent the best opportunity to exit the COVID-19 pandemic. As such, the goal of any vaccination campaign is to achieve vaccine-induced herd immunity as quickly as possible in order to prevent further transmission of disease [1]. However, data suggest a level of hesitancy among individuals when determining whether or not to receive a COVID-19 vaccine—a hesitancy that extends from childhood [2] and adult vaccines [3] to the novel H1N1 influenza pandemic [4] and COVID-19 pandemic vaccines [5]. Research indicates that COVID-19 vaccine hesitancy occurs across a heterogeneous group of individuals based on sex, race, ethnicity, and country of origin [6,7,8,9]. In Texas, the average rate of full-dose vaccination for COVID-19 was 62.4% after nearly 20 months of vaccine availability [10].

Vaccine hesitancy is a complicated construct. It involves attitudes that are specific to the person, place, time, and type of vaccine [11]. To better understand vaccine hesitancy as it relates to the complex interplay of personal, social, and cultural cues experienced by individuals, it is important to collect data locally. Understanding why people are vaccine-hesitant can support health education and health-promotion activities and encourage individuals to become vaccine acceptors instead of vaccine refusers [12].

In a study published in 2021, we reported on data collected from a cross-section of individuals in Central Texas to identify sociodemographic factors associated with COVID-19 vaccine hesitancy immediately prior to the release of the COVID-19 vaccine [9]. These factors included female sex, being of Black or African-American race, being aged 35–49 years old, having an annual income of less than USD 10,000, and having an education of less than a four-year college degree. That study, however, did not address the central tenet of why a person plans or does not plan to be vaccinated with one of the currently available COVID-19 vaccines. 

The COVID-19 Vaccination Uptake Behavioral Science Task Force for the US Centers for Medicare and Medicaid Services developed a framework to assess vaccine hesitancy [12]. The purpose of this framework was to address how COVID-19 vaccine uptake among long-term care facility employees could be increased. Briefly, the model stratified long-term care facility employees among three categories of vaccine uptake: (1) vaccine acceptors, (2) vaccine refusers, and (3) the moveable middle. Vaccine acceptors are individuals who have agreed to receive a vaccine and can potentially act as positive influencers and ambassadors to those who have not yet decided to receive a COVID-19 vaccine. Vaccine refusers are those who have indicated that they will not receive a COVID-19 vaccine and can potentially act as negative influencers to those who may be undecided. The moveable middle is a group of individuals who have not yet decided whether to receive a COVID-19 vaccine, representing individuals who may become either vaccine acceptors or vaccine refusers.

To better understand the reasons for vaccine hesitancy, we asked a cohort of individuals with health insurance offered as part of the Affordable Care Act (ACA) from Sendero Health Plans (Sendero) to tell us whether they planned to receive one of the newly available COVID-19 vaccines, and to tell us why they made this decision. This assessment occurred during the first week of public mRNA COVID-19 vaccine availability for people living in Austin, Texas. We report on qualitative feedback from Sendero Health Plan members with regard to their intent to receive a COVID-19 vaccine using a general inductive approach that reflects four COVID-19 vaccine uptake options as applied to the COVID-19 Vaccination Uptake Behavioral Science Task Force framework.

## 2. Materials and Methods

We conducted two cross-sectional surveys of individuals with health insurance living in Central Texas. All adult individuals (aged 18 years or older) who were head-of-household members of Sendero Health Plans were eligible to participate in Survey 1. Head-of-household members were defined as adult members aged 18 years or older who were the primary policyholder. Survey 1 collected sociodemographic data and information related to the COVID-19 pandemic. The details of Survey 1 are available elsewhere [9]. Individuals were eligible to participate in Survey 2 if they indicated in Survey 1 that they would be willing to answer additional questions related to the COVID-19 pandemic. Survey 1 was administered from 11 November 2020 to 22 December 2020, while Survey 2 was administered from 24 December 2020 to 31 December 2020. Individuals were invited by email to participate in Survey 2. All questions and communication were provided in English and Spanish.

All responses were submitted using the online platform Qualtrics (Qualtrics, Provo, UT, USA). Participation in Survey 2 was voluntary, and those who completed the survey were sent a USD 25 gift card for a local grocery merchant. All data were de-identified prior to analysis. Demographic variables of interest from Survey 1 included age, sex, race, ethnicity, household income status, and highest level of education. The Survey 1 demographics were matched to the Survey 2 respondents. This means that we collected demographic information related to race, ethnicity, sex, education, and annual household income in Survey 1, and we matched these responses to individuals in Survey 2. Additional questions of interest from Survey 2 included the following:

- “I plan to get the COVID-19 vaccine when it is available”. Categorical response options included “yes”, “no”, “prefer not to answer”, or “unsure”.
- “Please tell us why you answered [‘yes’, ‘no’, ‘prefer not to answer’, or ‘unsure’] to the statement, ‘I plan to get the COVID-19 vaccine when it is available’”.

A general inductive approach was used to analyze the qualitative variables to “allow research findings to emerge from the frequent, dominant, or significant themes inherent in [the] raw data, without the restraints imposed by structured methodologies” [13]. This approach follows Thomas’ methodology to create a brief summary of the raw data, establish links between the summary data and research objectives, and develop a framework of dominant domains evident from the data [13]. 

Two researchers independently reviewed, assessed, and coded each qualitative response to a specific theme. This information was entered into Microsoft Excel version 16.64 for Mac. After independent assignment of qualitative data to a theme, the two researchers conferred and formally defined the themes. Each response was assigned to only one theme. A total of 38 themes were identified. Further review of the 38 themes commenced, with assignment of each theme to one of eight domains. The domains represented the overall concept of the qualitative feedback statement. Responses that were not amenable to classification into an emerging theme or domain were classified as “not otherwise classified” and excluded from further analysis.

## 3. Results

A total of 737 persons completed the survey, with a response rate of 88.2%. Table 1 summarizes their demographic data. The mean respondent age was 48.2 years (range: 21.4–86.3, SD: ±11.8). Individuals identified as female comprised 54.0% of the respondents, and more than half of respondents (59.7%) reported having attained at least a bachelor’s degree. Slightly more than half of respondents (51.7%) reported a household income of less than USD 40,000 per year, with 38.3% of respondents reporting a household income of less than USD 30,000 per year. The majority of respondents self-identified as White (85.6%), while 18.9% self-identified as Hispanic, Latino, or Spanish. The participants in Survey 2 were of similar race and ethnicity as those who participated in Survey 1.

**Figure 1:**
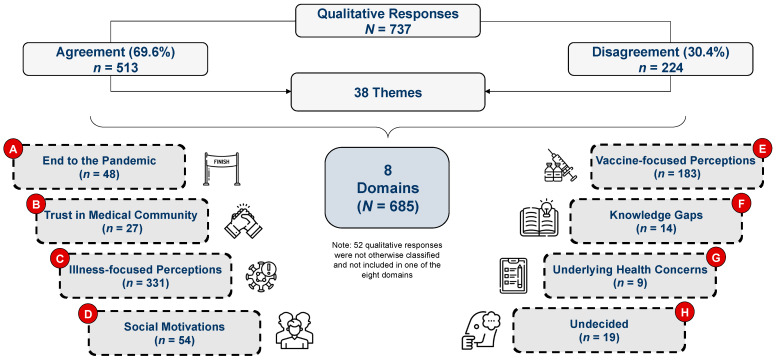
The inductive analytic process to stratify 685 responses into eight domains.

Two raters independently evaluated the qualitative responses and assigned 513 (69.6%) of the 737 qualitative responses to specific themes. The remaining 224 (30.1%) qualitative responses required concurrent review and discussion by both raters because the raters either did not agree on the assigned themes or were unable to identify an appropriate theme for assignment. For those 224 responses, the raters discussed and reviewed specific words in the responses, how these words aligned with responses already assigned to a particular theme, and whether any additional information in the response could provide information to assist the two raters in accurately assigning the response to an existing theme or a new theme. Altogether, 172 (76.7%) of the 224 remaining qualitative responses were assigned to an existing or new theme. The raters were unable to categorize 52 (23.2%) of the remaining 224 responses into a discernable thematic area; therefore, these responses were grouped as “not otherwise classified”. A total of 685 (93%) of the 737 original qualitative responses were therefore deemed valid and included in the qualitative analyses. The 38 themes were further consolidated into eight domains that best represented the conceptual elements of the data. Figure 1 summarizes the eight domains. 

**Figure 2:**
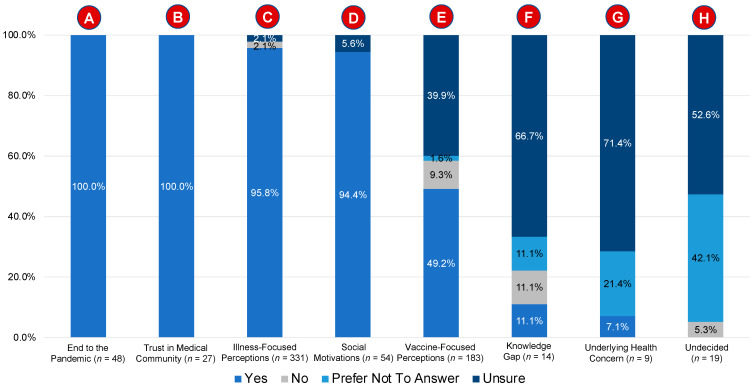
Proportion of individuals in each of the eight domains who responded “yes”, “no”, “prefer not to answer”, or “unsure” to the statement “I plan to obtain the COVID-19 vaccine when it is available”.

The majority of respondents (*n* = 535; 78.1%) said “yes” to the statement “I plan to obtain the COVID-19 vaccine when it is available”, while 3.8% (*n* = 26), 2.2% (*n* = 15), and 15.9% (*n* = 109) responded “no”, “prefer not to answer”, and “unsure”, respectively. The statement “I plan to obtain the COVID-19 vaccine when it is available” is hereafter referred to as the primary statement in the rest of this manuscript. Summary responses from each of the eight domains were stratified by the response to the primary statement (see Table 2). Figure 2 stratifies the responses to the primary statement by the eight domains. 

### 3.1. Domain A: End to the Pandemic (n = 48)

The responses from 48 individuals were mostly concerned with ending the pandemic. All responses in this domain were expressed by individuals who responded “yes” to the primary statement. For a response to be assigned to this domain, it had to relate directly to ending the pandemic or use words to that effect. Individuals who planned to receive a vaccine expressed a desire for the pandemic to end, for the world to get back to normal, and felt that the pandemic would only end if enough people were vaccinated (i.e., herd immunity). 

### 3.2. Domain B: Trust in the Medical Community (n = 27)

The responses from 27 individuals were mostly concerned with trust in the medical community and in the science responsible for vaccine development. All responses in this domain were expressed by individuals who responded “yes” to the primary statement. For a response to be assigned to this domain, it had to relate directly to trust in science, the medical community, or belief in the scientific process. Individuals who planned to receive a vaccine expressed belief in science, belief in vaccines, and trust in science and the guidelines. 

### 3.3. Domain C: Illness-Focused Perceptions (n = 331)

The responses from 331 individuals were mostly concerned with thoughts and concerns related to the COVID-19 illness. This was the largest single domain when measured by the number of responses. The majority of responses (*n* = 317; 95.8%) were from individuals who said “yes” to the primary statement. Seven (2.1%) and seven (2.1%) individuals responded “no” or “unsure”, respectively, to the primary statement. For a response to be assigned to this domain, it had to relate directly to the COVID-19 illness or infection. Individuals who planned to receive a vaccine believed they were at risk from COVID-19 infection, wished to avoid COVID-19 infection, and wanted to keep themselves safe. Individuals who responded “no” or “unsure” to the primary statement expressed concerns about whether they were at risk of disease and were somewhat skeptical as to the seriousness of the COVID-19 illness. 

### 3.4. Domain D: Social Motivations (n = 54)

The responses from 54 individuals were mostly concerned with social motivations related to ending the pandemic. The majority of responses (*n* = 51; 94.4%) were from individuals who said “yes” to the primary statement. Three (5.6%) individuals responded “unsure” to the primary statement. For a response to be assigned to this domain, it had to relate directly to a person’s perception of the disease from a group or community perspective. Individuals who planned to receive a vaccine wanted to protect themselves and others, felt the need to be vaccinated for work and travel reasons, and wanted to be able to visit elderly relatives. The three individuals in this domain who responded “unsure” to the primary statement were seemingly motivated by altruistic purposes, with each expressing empathy and concern for those who were more in need of the vaccine; as such, these three individuals indicated that they would receive a vaccine, but only after those more in need received it first.

### 3.5. Domain E: Vaccine-Focused Perceptions (n = 183)

The responses from 183 individuals were mostly concerned with thoughts and concerns related to the COVID-19 vaccines themselves. This was the second-largest single domain when measured by the number of responses. This domain was almost evenly split between individuals who said “yes” (*n* = 90; 49.2%) to the primary statement and those who responded “not yes” (*n* = 93; 50.8%)—as measured by “no”, “unsure”, or “prefer not to answer”—to the primary statement. The “unsure” group was the second-largest proportion of responders for this domain. For a response to be assigned to this domain, it had to relate directly to the COVID-19 vaccine or specifically reference the vaccine. Individuals who responded “yes” to the primary statement indicated their desire to receive the vaccine, their belief that the COVID-19 vaccine would work, and that they believed that the COVID-19 vaccine was important to obtain. 

Individuals who responded “no” (*n* = 17; 9.3%) or “unsure” (*n* = 73; 39.9%) to the primary statement were concerned about the vaccine, the technology, and the lack of research. Individuals who responded “prefer not to answer” (*n* = 3; 1.6%) to the primary statement expressed concerns related to vaccine safety and the need for more research on the vaccine before determining whether they would receive the vaccine.

### 3.6. Domain F: Knowledge Gap (n = 14)

The responses from 14 individuals were mostly concerned with a lack of knowledge about the vaccine. This was the second-smallest single domain when measured by the number of responses. The majority of responses (*n* = 13; 92.9%) were from individuals who responded “unsure” or “prefer not to answer” to the primary statement. One (7.1%) individual responded “yes” to the primary statement. For a response to be assigned to this domain, it had to relate directly to a lack of knowledge or a need for additional information and education. Individuals who responded “unsure” or “prefer not to answer” to the primary statement wanted more information about the vaccine before deciding whether or not be vaccinated.

### 3.7. Domain G: Underlying Health Concern (n = 9)

The responses from nine individuals were mostly concerned with underlying health issues. This was the smallest single domain when measured by the number of responses. Six (66.7%) individuals responded “unsure” to the primary statement, while the remaining three individuals responded either “yes”, “no”, or “prefer not to answer” (33.3%). For a response to be assigned to this domain, it had to relate directly to a specific health concern. Indeed, 100% of responses in this category mentioned or alluded to a specific individual health concern and a need for further information on how the COVID-19 vaccine may impact their specific health concern. Specific responses are not provided, in order to protect personal health information.

### 3.8. Domain H: Undecided (n = 19)

The responses from 19 individuals mostly reflected indecision on whether to receive a COVID-19 vaccine. One respondent responded “no” (5.3%), eight responded “prefer not to answer” (42.1%), and 10 responded “unsure” (52.6%) to the primary statement. For a response to be assigned to this domain, it had to relate directly to some degree of indecision about whether to receive a vaccine. Most responses included a direct or indirect reference to being undecided.

## 4. Discussion

The results from our study show patterns of COVID-19 vaccine uptake among vaccine acceptors, vaccine refusers, and individuals in the moveable middle who have not yet decided about their plans to get vaccinated. Vaccine acceptors are individuals who acknowledge that they plan to receive a COVID-19 vaccine, while vaccine refusers are individuals who do not plan to receive a COVID-19 vaccine. The moveable middle consists of individuals who have not yet decided about their plans to accept or refuse the COVID-19 vaccine and may, given appropriate information and support, become vaccine acceptors. 

### 4.1. Vaccine Acceptors

Vaccine acceptors made up 78.1% (*n* = 535) of respondents across seven domains. Two of these domains (A: End to the Pandemic and B: Trust in the Medical Community) were dominated by vaccine acceptors, with 100% of respondents saying that they planned to receive a COVID-19 vaccine. Both domains represent perceived benefits of vaccination; therefore, individuals who want to see the pandemic end and who have trust in the scientific method are more likely to plan to receive a vaccine. This finding is echoed in a qualitative study from Hong Kong, in which respondents who perceived the COVID-19 vaccines as conferring benefits were significantly more likely to accept the vaccines [14].

The Illness-Focused Perception domain (C) and the Social Motivations domain (D) represented high proportions of vaccine acceptors, at 95.8% and 94.4%, respectively. For Illness-Focused Perceptions (C), the susceptibility to and perceived severity of COVID-19 were primary motivators for vaccination—a finding that is consistent with the literature. For example, the Health Belief Model recognizes “perceived likelihood of harm if no action is taken and perceived seriousness of the consequences if harm was to occur” to support vaccination [15]. Recent qualitative research from Malaysia reported that 63.3% of respondents expressed a desire to receive a COVID-19 vaccine when perceived susceptibility to disease was high, with “prevention is better than cure”, “immunity”, and a “negative perception of COVID-19” as subthemes related to this Health Belief Model construct [16].

In the Social Motivations domain (D), the primary motivator for vaccination was for the good of the community and a desire to participate in community activities, whether for work, travel, or interaction with family members. As such, the Social Motivations domain (D) exhibited a perceived benefit to vaccination in order to permit social interaction once again. Domain E represents Vaccine-Focused Perceptions. Individuals who believed that there was a benefit to receiving a COVID-19 vaccine represented just under 50% of respondents in this domain. 

### 4.2. Vaccine Refusers

Vaccine refusers were a cohort of 26 individuals who responded “no” to the primary statement. None of the domains clearly represented vaccine refusers. Rather, the 26 persons who responded “no” were a heterogeneous group represented by four domains: Illness-Focused Perceptions (C), Vaccine-Focused Perceptions (E), Underlying Health Concerns (G), and Undecided (H). The majority of “no” respondents (65.4%; 17 of 26) were represented by the Vaccine-Focused Perceptions domain (E). Interestingly, upon examination, most of the “no” responses across the four domains (C, E, G, and H) were more suggestive of being in the moveable middle than being true vaccine refusers. Thus, while these individuals responded “no” to the primary statement, their qualitative feedback indicates something different—that they may indeed be temporary “no” respondents, pending additional information and education. Qualitative feedback from “no” respondents who may be thought of as being part of the moveable middle included the following:

- *“Way to [sic] risky given how little testing was done”.*
- *“Because there is not enough research about long-term side effects”.*
- *“I would like to see the data after 12 months of administering the vaccine globally before considering it myself”.*
- *“There is not enough information available on the side effects of the vaccine”.*
- *“With the mRNA technology being so new, I want to see further data. I also want to see the effective rate in the real world as opposed to the lab”.*
- *“Need more human testing”.*

These representative statements from the “no” group, while currently indicating no desire to be vaccinated, seem to allow an opportunity for vaccine acceptance if their concerns can be addressed.

True vaccine refusers are thought to represent about 2–3% of the population [12], but real-world evidence to this effect is scarce. In a separate study by our research team, we tried to disentangle true vaccine refusers from those who may, in fact, be in the moveable middle despite having responded “no” when asked whether they planned to receive a COVID-19 vaccine [17]. In that study, we defined true vaccine refusers as persons who (1) had not received a vaccine as of July 2021, (2) did not plan to receive a vaccine, and (3) when asked said that “nothing could be done to change their mind to obtain the COVID-19 vaccine”. Our data indicate that the proportion of individuals who met these three criteria and who could be deemed true vaccine refusers, or never-evers, was 2.1%, or 19 out of 900 respondents.

### 4.3. Moveable Middle

The moveable middle (*n* = 124) was represented by six of the eight domains, not including Domains A (End to the Pandemic) and B (Trust in the Medical Community). The underlying concept of the moveable middle identified three distinct subgroups: the “make it easy” subgroup, the “influence and boost motivation” subgroup, and the “build trust in vaccine safety” subgroup. 

The “make it easy” subgroup posits that individuals need improved access in order for them to obtain the vaccine. In our data, we found a handful of respondents who indicated a concern about vaccine access and logistics, corresponding to the “make it easy” group. Such concerns were expressed in the Social Motivations domain (D). Interestingly, the responses were altruistic in nature, with a preference for those more in need to receive the vaccines first due to limited quantities at the onset of vaccine distribution. Some of the feedback included “Want the most in need to get it first. Front line workers/elderly/etc.”, “I will wait for others that I know to get it first”, and “I want the most vulnerable to get it first”. Interestingly, at the time that the survey was available for completion, COVID-19 vaccine availability was limited, but limited access ceased to be an issue after about six months. However, despite limited vaccine availability when individuals completed this survey, access was a minor, altruistic issue.

The “influence and boost motivation” subgroup posits that social influence, communication, and motivation can motivate individuals to become vaccine acceptors. Overall, we did not identify responses in our data that were reflective of this subgroup, except for a few comments related to seeking and waiting on advice from a physician. However, such influencers did not extend beyond health or medical professionals to other key opinion leaders in the community.

The “build trust in vaccine safety” subgroup posits that increased education and closing the knowledge gap, particularly around vaccine safety and efficacy is needed for individuals to plan to obtain the vaccine. The majority of comments from our moveable middle cohort fell within the “build trust in vaccine safety” subgroup. Representative comments included the following:

- *“I need more information and time to decide”.*
- *“Tendría que platicarles con mi doctor”. [I would have to talk to my doctor.]*
- *“Because I’m not sure just yet but I know I need it due to my medical condition”.*
- *“I’m just not completely sure about how I feel about the safety and efficacy”.*
- *“Would like additional long term data published”.*
- *“I’d like to see how others respond to the vaccine before I get it”.*

### 4.4. Limitations

This study has several limitations. Firstly, this survey was completed during the first week of mRNA vaccine availability in Austin, Texas. At that time, the vaccines were still new, were only approved under Emergency Use Authorization, and had only begun to be administered to high-risk groups based on the US Centers for Disease Control and Prevention guidelines. Over time, individual concerns about the vaccine approval process and vaccine safety may have changed.

Secondly, individuals may have been reluctant to provide detailed health information to their health insurance company, particularly if this information could be perceived as a risky health behavior (e.g., not obtaining the COVID-19 vaccine). 

Thirdly, the theoretical framework used in this study was developed for workers in the long-term care setting. We are not aware of any studies to date applying this framework to the general public.

Fourthly, vaccine acceptors represented 78.1% (*n* = 535) of the respondents to this survey, the majority of whom lived in Travis County. This high acceptance rate may be partially explained by the urban–rural divide in Texas. Based on data from the Texas Department of State Health Services, we know that individuals living in urban counties tend to have a higher COVID-19 vaccination rate than individuals living in rural counties. For example, Travis County, Texas, which is urban and includes the state capital of Austin, has a full-dose vaccination rate of 70.7%. This is considerably higher than the seven rural East Texas counties that make up the Northeast Texas Public Health District of Gregg (47.9%), Smith (47.7%), Wood (44.4%), Anderson (42.8%), Henderson (41.0%), Van Zandt (38.4%), and Rains counties (38.2%), none of which have attained a 50% vaccination rate [18]. 

The final limitation involves the sample itself. The pool of individuals eligible to participate in this survey consisted of individuals who had purchased health insurance on the individual marketplace. As such, these individuals are likely to exhibit health-seeking behaviors. While we are not aware of specific biases from these individuals, we expect that individuals who purchase health insurance are more likely to exhibit health-seeking behaviors and, therefore, may be overrepresented as vaccine acceptors.

### 4.5. Recommendations

We believe that this research has practical application when preparing for future pandemics that involve vaccine-preventable diseases, including the following:

1. In the event of a future pandemic with potential for vaccine mitigation, local health departments should immediately assess potential hesitancy in their community in order to delineate between vaccine acceptors, the moveable middle, and vaccine refusers. It is important to collect both quantitative and qualitative data, with the latter directly asking whether a person plans to receive the vaccine when it becomes available.
2. Education and outreach can then be developed to address the concerns of people who represent the moveable middle and may be amenable to becoming vaccine acceptors. The goal is to learn as early as possible why someone may be vaccine-hesitant, and to develop ways to engage with and address the very real concerns that they may have.

## 5. Conclusions

These data provide insight from 685 persons on their decision-making process across the complex interplay of place (i.e., Central Texas), time (i.e., when COVID-19 vaccines first became available locally), and vaccine (i.e., the Moderna and Pfizer mRNA vaccines) related to COVID-19 vaccine hesitancy. We report on these data because it is important to understand why individuals made the decision they did regarding plans to receive the COVID-19 vaccines. It is also important to identify lessons learned from their decision-making processes and to improve public health emergency preparedness planning and response operations to support vaccine acceptance in case of future pandemics. 

Vaccine uptake for a pandemic is strongest in the earliest months of vaccine availability. The feedback provided in this study is a first step to using a data-driven approach to help prepare education and messaging to address concerns about vaccine hesitancy early in a pandemic, when uptake is most likely to occur. These data show clear delineations between vaccine acceptors, vaccine refusers, and the moveable middle across eight domains that can assist public health professionals in developing information to respond to the information needs of the public as they make the very personal choice about whether to receive a vaccine.

Finally, additional research is needed to better delineate the proportion of true vaccine refusers, or the never-ever group. Disentangling the never-ever group from those who have temporarily said “no” is important from a public policy perspective. The education and outreach to these two groups will likely differ, and one could posit that education and outreach targeted to the never-ever group may even have a limited effect, and that these resources could be better focused on those who may genuinely be in the moveable middle.

## Figures and Tables

**Table 1:** Reported demographic and summary characteristics of the survey respondents.

Characteristics of the Respondent Population	N (%)
Sex	737 (100)
Female	398 (54.0)
Male	339 (46.0)
Age in years	
18–24 years old	9 (1.2)
25–34 years old	121 (16.4)
35–44 years old	173 (23.5)
45–54 years old	168 (22.8)
55–64 years old	261 (35.4)
≥65 years old	5 (0.7)
Race *	737
American Indian or Alaskan Native	11 (1.5)
Asian	41 (5.6)
Black or African-American	38 (5.2)
Native Hawaiian or other Pacific Islander	3 (0.4)
White	631 (85.6)
Other	45 (6.1)
Ethnicity	737
No, not of Hispanic, Latino, or Spanish origin	598 (81.1)
Yes, Hispanic, Latino, or Spanish origin	139 (18.9)
Education	737
Some high school	19 (2.6)
High school diploma, GED, or equivalent	64 (8.7)
Trade school	25 (3.4)
Some college	132 (17.9)
Associate degree	52 (7.1)
Bachelor’s degree	282 (38.3)
Graduate degree	158 (21.4)
Other	5 (0.7)
Annual household income	737
Less than USD 10,000 per year	67 (9.1)
USD 10,000–29,999	215 (29.2)
USD 30,000–39,999	99 (13.4)
USD 40,000–49,999	67 (9.1)
USD 50,000–75,999	110 (14.9)
USD 76,000–99,999	47 (6.4)
USD 100,000 or above	64 (8.7)
Prefer not to answer	59 (8.0)
Other	9 (1.2)

* Respondents were able to represent their racial heritage by selecting more than one racial group; therefore, the total *n* for the race variable may be greater than 737.

**Table 2:** Qualitative feedback for each domain stratified by response type: “yes”, “no”, “prefer not to answer”, or “unsure”.

Domain (N)	Response *	*n* (%)	Representative Qualitative Feedback
A. End to the Pandemic (N = 48)	Yes	48 (100%)	Vital to control Covid! Because it want [sic] this pandemic to end! And I wish to be part of the solution, not the problem. Creo que es necesario que todos estemos vacunados para bajar el impacto de la pandemia [I think it is necessary for all of us to be vaccinated to reduce the impact of the pandemic]. Everyone needs to get the vaccine so we can go back to something resembling normal. The only way to reduce/eliminate it will be herd immunity, which is best reached through immunization.
	No	-	[No responses for this category]
	Unsure	-	[No responses for this category]
	PNTA *	-	[No responses for this category]
B. Trust in the Medical Community (N = 27)	Yes	27 (100%)	I believe in science and the pandemic will continue if the world doesn’t develop immunity. I trust the people who say it’s safe to take and believe that the more people who get it the safer everyone will be. Science, reason, logic and data. I believe in science and this is our only hope to end pandemic. I believe in science and the vaccines are looking good!
	No	-	[No responses for this category]
	Unsure	-	[No responses for this category]
	PNTA	-	[No responses for this category]
C. Illness-Focused Perceptions (N = 331)	Yes	317 (95.8%)	It’s important to me to get vaccinated in order to prevent myself and those around me from contracting the pandemic virus. I want to do everything possible to help protect my health and the general public health. I want to be protected and help slow the spread. I want the best chances of being protected from the disease. Scared of COVID.
	No	7 (2.1%)	I don’t think I need it as badly as others. I don’t know if I’ll need it. 99.99% survival rate. No one needs the covid vaccine. Gotta make that pharmo [sic] company rich somehow. The statistics which I have read suggest that I am not likely to have severe, if any, symptoms when exposed to COVID-19. I am going to see how it all pays [sic] out. I am not worried about covid-19 because I don’t have any pre-existing conditions that would make it dangerous to me.
	Unsure	5 (1.5%)	
	PNTA	-	[No responses for this category]
D. Social Motivations (N = 54)	Yes	51 (94.4%)	I want to add to the progression of our society post covid. I’d like to be vaccinated so I can see my friends and family again and travel safely. Mother’s Nursing Home requires it and offers it. Probably need vaccine to be employable.
	No	-	[No responses for this category]
	Unsure	3 (5.6%)	[Data not provided because *n* < 5 for this category]
	PNTA	-	[No responses for this category]
E. Vaccine-Focused Perceptions (N = 183)	Yes	90 (49.2%)	The COVID shot is important Vaccines are important not only for myself but for my family, friends, and neighbors I always get vaccinated when needed Seems like the pertinent thing to do. I always get vaccinated
	No	17 (9.3%)	I will wait for the 1st round of people to die from such a short testing time. Way to [sic] risky given how little testing was done. With the mRNA technology being so new, I want to see further data. I also want to see the effective rate in the real world as opposed to the lab. Untested. Because I not I think [sic] that the vaccines need more studying.
	Unsure	73 (39.9%)	Will wait to see how the vaccine progresses. I think more testing is needed. I want to see the results of those initially vaccinated. Still weighing the options of the side effects versus the virus there’s not enough known about the vaccine yet in my opinion. Unsure of any long term side effects since it was approved so quickly.
	PNTA	3 (1.6%)	[Data not provided because *n* < 5 for this category]
F. Knowledge Gap (N = 14)	Yes	1 (7.1%)	[Data not provided because *n* < 5 for this category]
	No	-	[No responses for this category]
	Unsure	13 (92.9%)	I am still researching. Honestly, I figure by the time that vaccine gets around to me, there will be a lot more known about Covid-19 and the vaccine. I’ll make the decision once it’s available to me based on the most current information when that time comes. Tendría que platicarles con mi doctor [I would have to talk to my doctor]. Don’t know much about the vaccine to determine if I will for sure get it. I need more information and time to decide.
	PNTA	-	[No responses for this category]
G. Underlying Health Concern (N = 9)	Yes	1 (11.1%)	Because the responses reflected individual concern about specific health conditions, specific responses are not provided.
	No	1 (11.1%)	
	Unsure	6 (66.7%)	
	PNTA	1 (11.1%)	
H. Undecided (N = 19)	Yes	-	
	No	1 (5.3%)	[Data not provided because *n* < 5 for this category]
	Unsure	10 (52.6%)	Because I’m not sure I’m not sure I will get the vaccine Haven’t thought about it yet Just haven’t made up my mind yet. No specific reason, just not sure if I’m going to
	PNTA	8 (42.1%)	Undecided Because I’m not sure what I want to do I haven’t decided yet

* PNTA, Prefer Not To Answer.

## Data Availability

De-identified data can be requested from the corresponding author.
